# Lineage tracing reveals evidence of a popliteal lymphatic muscle progenitor cell that is distinct from skeletal and vascular muscle progenitors

**DOI:** 10.1038/s41598-020-75190-7

**Published:** 2020-10-22

**Authors:** H. Mark Kenney, Richard D. Bell, Elysia A. Masters, Lianping Xing, Christopher T. Ritchlin, Edward M. Schwarz

**Affiliations:** 1 Center for Musculoskeletal Research, University of Rochester Medical Center, Box 665, 601 Elmwood Ave, Rochester, 14642 NY USA; 2 Department of Pathology & Laboratory Medicine, University of Rochester Medical Center, Rochester, NY USA; 3 Department of Biomedical Engineering, University of Rochester, Rochester, NY USA; 4 Division of Allergy, Immunology, Rheumatology, Department of Medicine, University of Rochester Medical Center, Rochester, NY USA; 5 Department of Orthopaedics, University of Rochester Medical Center, Rochester, NY USA

**Keywords:** Mesenchymal stem cells, Muscle stem cells

## Abstract

Loss of popliteal lymphatic vessel (PLV) contractions, which is associated with damage to lymphatic muscle cells (LMCs), is a biomarker of disease progression in mice with inflammatory arthritis. Currently, the nature of LMC progenitors has yet to be formally described. Thus, we aimed to characterize the progenitors of PLV-LMCs during murine development, towards rational therapies that target their proliferation, recruitment, and differentiation onto PLVs. Since LMCs have been described as a hybrid phenotype of striated and vascular smooth muscle cells (VSMCs), we performed lineage tracing studies in mice to further clarify this enigma by investigating LMC progenitor contribution to PLVs in neonatal mice. PLVs from Cre-tdTomato reporter mice specific for progenitors of skeletal myocytes (Pax7^+^ and MyoD^+^) and VSMCs (Prrx1^+^ and NG2^+^) were analyzed via whole mount immunofluorescent microscopy. The results showed that PLV-LMCs do not derive from skeletal muscle progenitors. Rather, PLV-LMCs originate from Pax7^−^/MyoD^−^/Prrx1^+^/NG2^+^ progenitors similar to VSMCs prior to postnatal day 10 (P10), and from a previously unknown Pax7^−^/MyoD^−^/Prrx1^+^/NG2^−^ muscle progenitor pathway during development after P10. Future studies of these LMC progenitors during maintenance and repair of PLVs, along with their function in other lymphatic beds, are warranted.

## Introduction

The lymphatic system functions to shuttle immune cells and regulate interstitial fluid environments by pumping lymph throughout the body via a series of lymphatic vessels and lymph nodes^1,2^. The lymphatic vasculature consists of three predominant segments with important structural differences: capillaries, pre-collecting, and collecting lymphatic vessels (CLVs). Lymphatic capillaries are blind ended vessels that consist of a lymphatic endothelial cell (LEC) monolayer and reside within the interstitial space where extracellular fluid flows down a pressure gradient to be absorbed into the lumen of the permeable lymphatic capillary bed. The lymph then flows through pre-collecting vessels with minimal lymphatic muscle cell (LMC) coverage and eventually transitions into the larger CLVs completely surrounded by LMCs. The CLVs actively move lymph on a one-way route to the cardiovascular system through a series of contractions^1,3^. Similar to veins, lymphatic vessels utilize surrounding skeletal muscle contractions as a major contributor to physically move fluid throughout the vasculature^4^. However, unlike the large vessels of the cardiovascular system, CLVs do not rely on a central pump, such as the heart, to move fluid in the absence of skeletal muscle action. Instead, the LMCs that surround CLVs contain unique contractile mechanisms that apply both an intrinsic pulsatile force to move lymph in distinct fluid boluses and provide tone to modulate lymphatic flow rates, while VSMCs primarily mediate tone to control cardiac-derived fluid flow^5^. Towards characterizing these distinct LMCs, it was previously discovered that LMCs possess an enigmatic actin isotype profile consisting of both vascular alpha-smooth muscle actin (αSMA) and striated (skeletal and/or cardiac) actin isoforms^6^. Additionally, various studies have focused on ex vivo contractile regulation of LMCs exposed to particular environmental stimuli or pharmacologic agents to determine the mechanistic underpinnings of lymphatic contractions^7^. Importantly, the physiologic function of lymphatic contractions is essential to promote homeostasis and prevent disease.

Lymphatic vessels have also been studied in disease^8^. In the case of inflammatory arthritis, we have demonstrated that lymphatic contractile dysfunction progresses over time in popliteal lymphatic vessels (PLVs) draining the ankle joint of tumor necrosis factor-transgenic (TNF-Tg) mice^9,10^. In the early phase of arthritis, PLVs sustain their contraction frequency in the short term and infiltrating immune cells travel from the inflamed ankle to promote popliteal lymph node (PLN) expansion. While lymphatic flow limits disease progression initially, PLVs become overwhelmed and vessel contractions cease concomitant with PLN collapse. These dramatic lymphatic functional changes are accompanied by an atrophic and apoptotic PLV-LMC phenotype at the ultrastructural level^11^. The loss of PLV contractions is associated with severe arthritic disease presumably because inflammatory infiltrates are confined to the joint resulting in prolonged synovitis along with enhanced bone and cartilage erosion^12–15^. Remarkably, anti-TNF therapy in TNF-Tg mice with late-stage disease restores PLV contractions concomitant with amelioration of arthritis^11^. As outlined above, the maintenance of functional LMCs is critical for joint homeostasis, so fundamental discoveries into the origin and function of LMC progenitors is required to fully understand the therapeutic potential of LMC renewal in inflammatory arthritis, and other disease states with CLV dysfunction.

**Figure 1:**
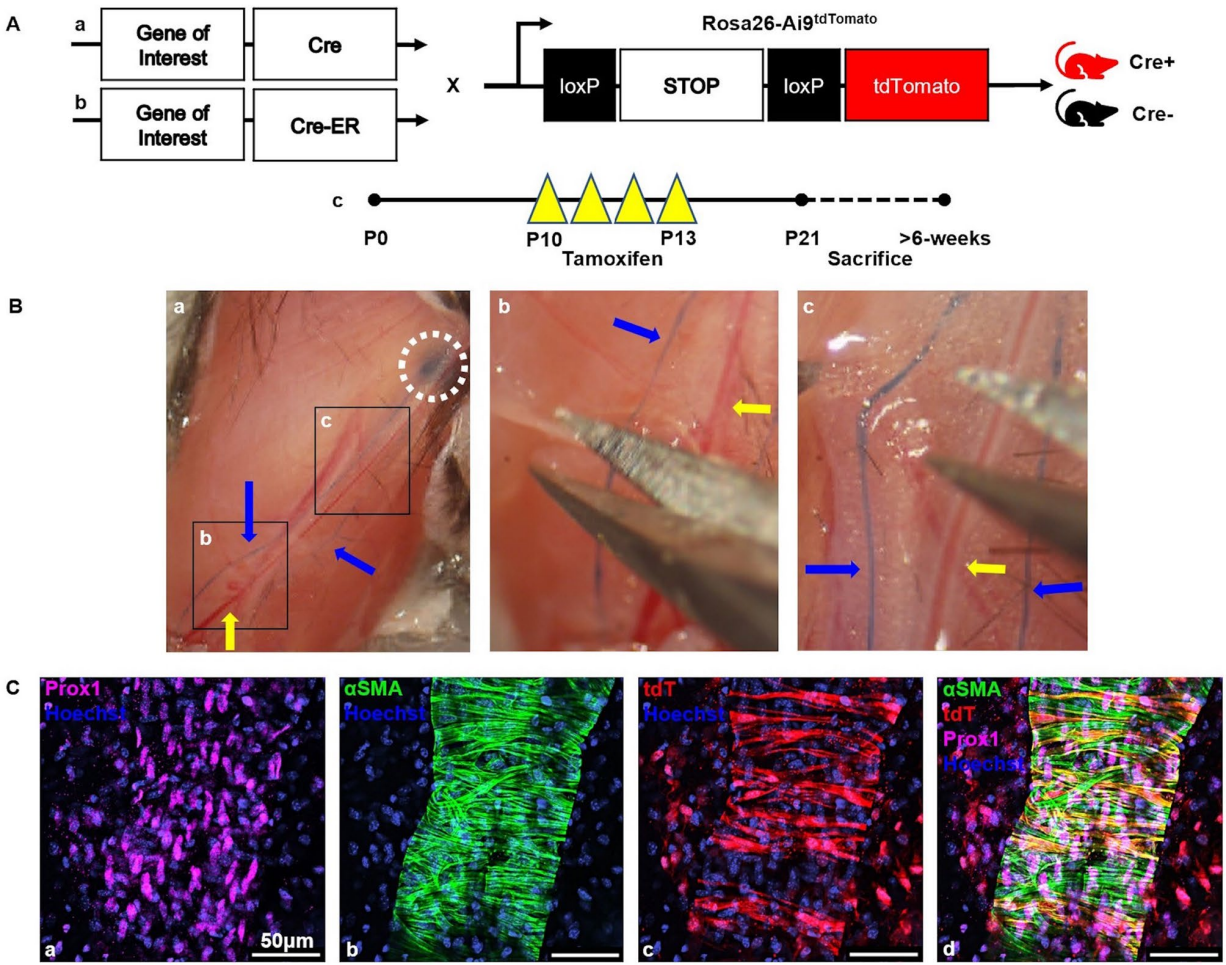
Genetic models for lineage tracing lymphatic muscle cells in popliteal lymphatic vessels and ex vivo whole mount multicolor fluorescent microscopy. Schematics of the constitutively-active (**A.a**) and tamoxifen-inducible (**A.b**) conditional Cre-driver constructs used in this study (Genes of Interest = Pax7, MyoD, Prrx1, and NG2) are presented with the Ai9^tdTomato^ reporter (tdT) construct to illustrate the heterozygous double-transgenic mice that were used in the lineage tracing (red Cre^+^), and their heterozygous single-transgenic Ai9^tdTomato^ littermates that were used as negative controls (black Cre^−^). An illustration of the tamoxifen dosing regimen (daily intraperitoneal injections on postnatal days 10–13) is also presented with the standard time of sacrifice at postnatal day 21, while all gene markers that showed PLV-LMC tdT-negativity at any time during neonatal development were additionally sacrificed > 6-weeks to validate earlier observations (**A.c**). To harvest the popliteal lymphatic vessels (PLVs), mice were anesthetized and injected with 2% Evans Blue dye (Millipore Sigma Cat# E2129) into their footpad, prior to skin removal. A 1.5 × photograph of the exposed lower limb is presented to illustrate how Evans Blue filled PLVs (two PLVs per hindlimb; blue arrows) draining into the popliteal lymph node (white circle) for PLV identification prior to excision (**B.a**). Magnified photographs of the black boxed regions illustrate incisions on the PLV border opposite the saphenous vein (yellow arrow) to release it from the fat pad (**B.b**), and then how the PLV was removed +/− the saphenous vein for analysis (**B.c**). After explant of the PLV (~ 2–3 mm in length), whole mount immunofluorescence was performed using antibodies against Prox1 with a DyLight 650-conjugated secondary antibody to mark lymphatic endothelial cells (purple) (**C.a**) and directly adjacent Alexa Fluor 488-conjugated antibodies against αSMA to mark lymphatic muscle cells (green) (**C.b**) with a Hoechst nuclear stain (blue). Direct fluorescent microscopy of the Cre-driven tdT reporter protein (red) in cells of the PLV (example of Prrx1^CreER^ × Ai9^tdTomato^ mouse) identifies positive cells in the lineage trace (**C.c**). A composite overlay of the three images is shown to illustrate co-expression of αSMA and tdT (yellow) in LMCs of Prox1^+^ PLVs (**C.d**).

Herein, we perform lineage tracing studies with Cre-tdTomato (tdT) reporter mice to determine the PLV-LMC progenitor cell pathway during neonatal development. Given the combined striated and vascular smooth muscle cell (VSMC) characteristics of LMCs^6^, we utilized markers involved in the differentiation of both these muscle cell types. Since the PLVs reside directly atop skeletal muscle, and are quite distant from the heart, we focused on the skeletal myocyte component of striated muscle cells. To assess skeletal progenitor origin, we utilized Cre-drivers for Pax7^Cre^^16^ and MyoD^iCre^^17^ as known transcription factors involved in skeletal myoblast differentiation. Additionally, to compare the transcriptional profile of VSMC progenitors, we used Cre-drivers for Prrx1^Cre/CreER^^18,19^ and NG2^Cre/CreER^^20,21^ which are known mesenchymal progenitor cell markers^22^ (see Methods for a detailed description of the gene markers). To assess transcriptional markers of PLV-LMC progenitors in early (before postnatal day 10 (P10)) and late (after P10) neonatal development, we utilized constitutive and tamoxifen-inducible Cre models, respectively (Fig. 1A). We performed PLV dissections as described in Fig. 1B, and all PLVs were analyzed by whole mount multicolor fluorescent microscopy with immunostaining for αSMA (LMCs) and Prox1 (lymphatic endothelial cells (LECs)). The main outcome measure was the presence or absence of αSMA^+^/tdT^+^ LMCs on PLVs defined by Prox1^+^ LECs (Fig. 1C). Our results reveal that PLV-LMCs derive from two distinct genetic lineages during neonatal development: Pax7^−^/MyoD^−^/Prrx1^+^/NG2^+^ progenitors prior to P10, and Pax7^−^/MyoD^−^/Prrx1^+^/NG2^−^ progenitors after P10 during later stages of neonatal growth and remodeling.

## Results

### PLV-LMCs do not derive from skeletal muscle progenitor cells during development

**Figure 2:**
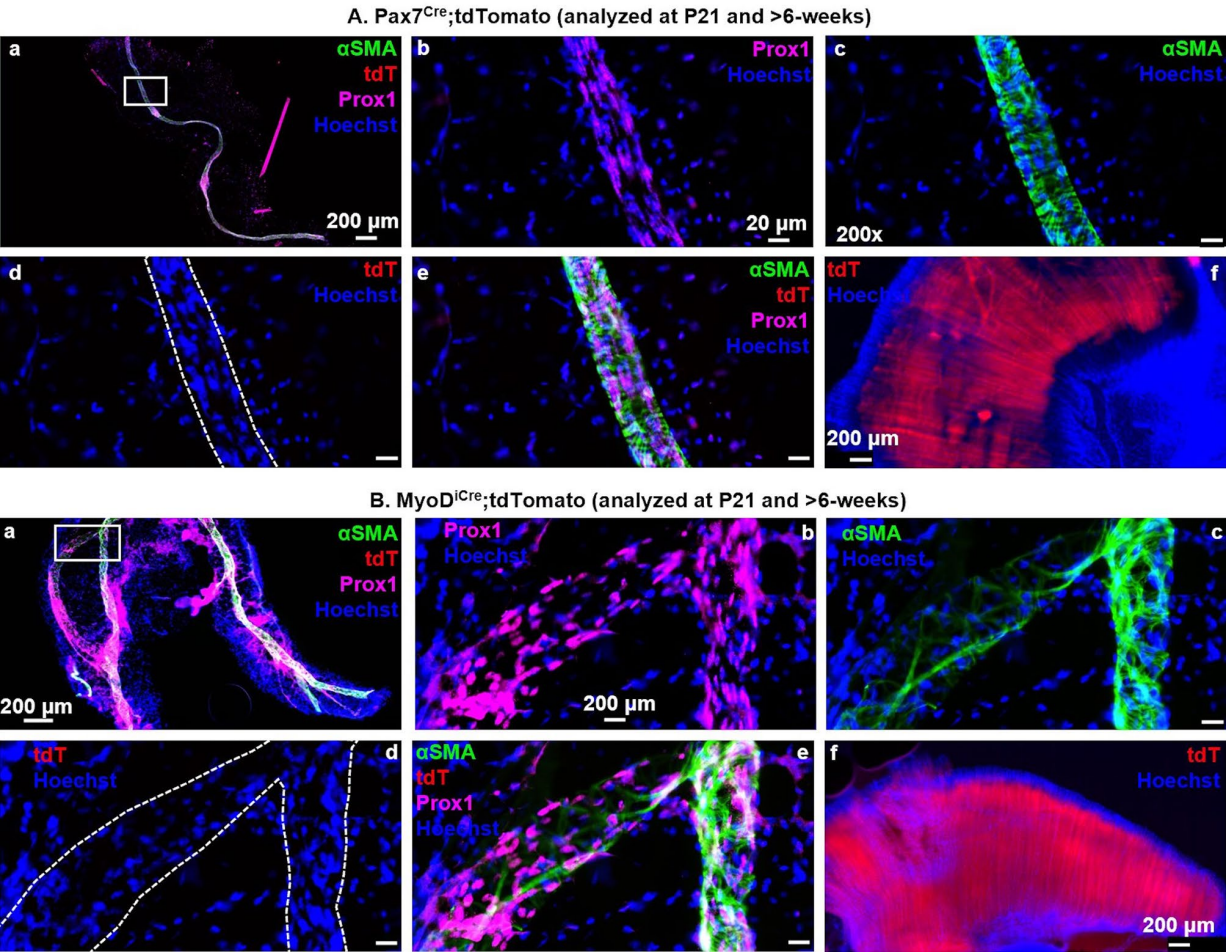
PLV-LMCs do not derive from skeletal muscle progenitor cells during development. Multicolor fluorescent microscopy was performed on whole mount immunostained PLVs from constitutive Pax7^Cre^ (n = 7, P200 depicted) and MyoD^iCre^ (n = 3, P21 depicted) reporter mice as described in Fig. 1, and direct fluorescent microscopy was performed on Hoechst stained diaphragm tissue from the same muscle-reporter transgenic mice as a positive control. Representative low-magnification images of PLVs with a highlighted region of interest (white boxes) (**A.a**,**B.a**) are shown with corresponding high-magnification images of Prox1 immunostain of LECs (**A.b**,**B.b**), αSMA immunostain of LMCs (**A.c**,**B.c**), the Cre-driven tdT expression (note lack of red fluorescence within the white dotted lines that outline the PLV) (**A.d**,**B.d**), and overlay of all channels (**A.e**,**B.e**). Representative images of diaphragm tissue are shown to illustrate the robust Cre-driven tdT expression in skeletal myocytes from these mice (**A.f**,**B.f**).

To test if PLV-LMCs originate from skeletal muscle progenitors, we utilized constitutive Cre-drivers for Pax7 and MyoD, with analysis at P21. In addition, we assessed LMC contribution in both strains after 6-weeks to validate the early findings in P21 mice. The results showed no evidence of tdT expression colocalizing with αSMA^+^ PLV-LMCs from Pax7^Cre^ animals at P21 or after 6-weeks (Fig. 2A.a–e, P200 PLVs depicted). Consistently, we also found no tdT colocalization with αSMA^+^ PLV-LMCs in MyoD^iCre^ mice at either time point (Fig. 2B.a–e, P21 depicted). Successful Cre-driven tdT expression was demonstrated in positive control skeletal myocytes of the diaphragm in both models (Fig. 2A,B.f). Thus, we find that PLV-LMCs arise from Pax7^−^/MyoD^−^ progenitors distinct from typical skeletal myocyte origin.

### PLV-LMCs originate from a Prrx1^+^ and NG2^+^ progenitor cell similar to VSMCs prior to P21

**Figure 3:**
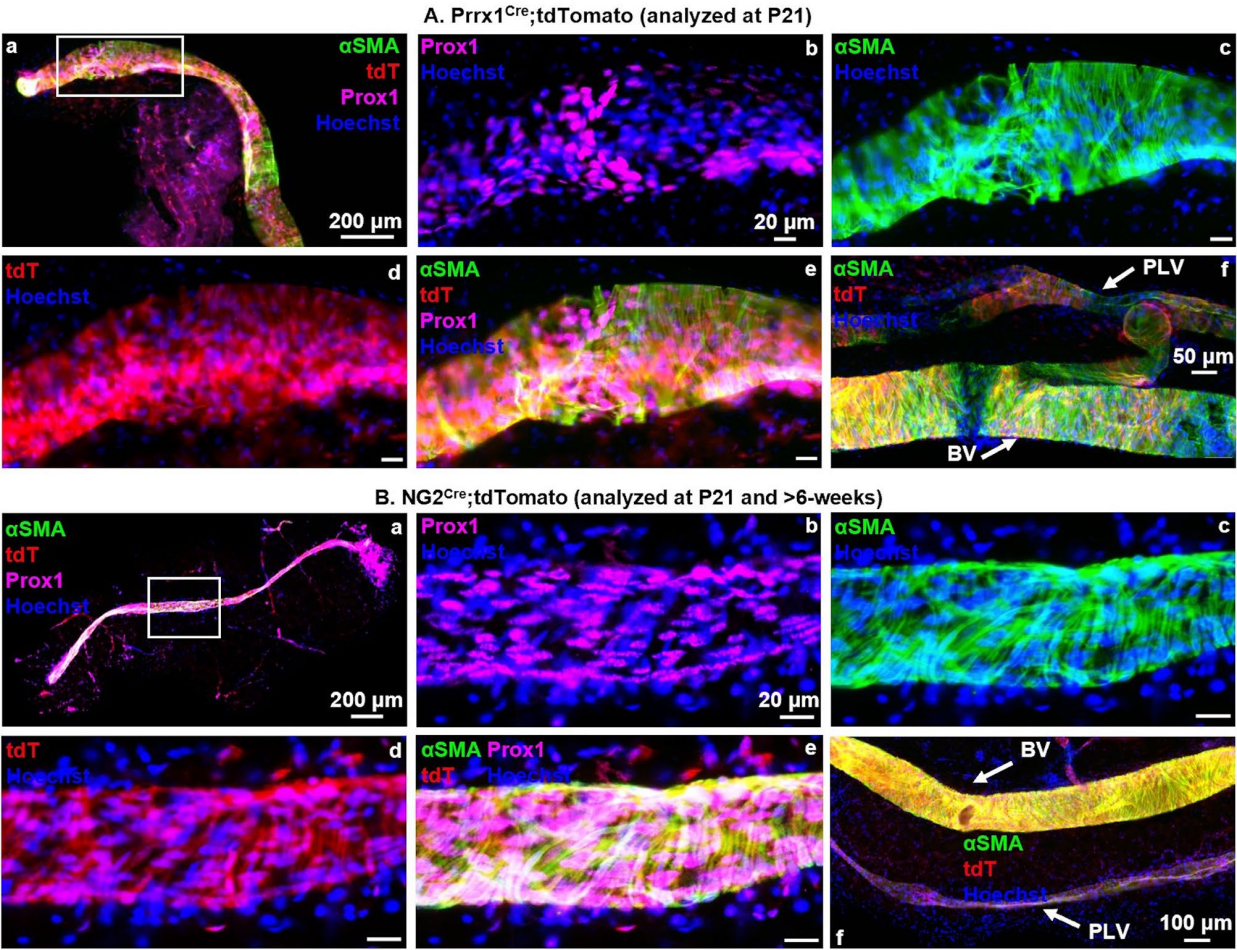
PLV-LMCs originate from a Prrx1^+^ and NG2^+^ progenitor cell similar to VSMCs prior to P21. Multicolor fluorescent microscopy was performed on whole mount immunostained PLVs and adjacent blood vessels (BVs) from constitutive Prrx1^Cre^ (n = 3, P21 depicted) and NG2^Cre^ (n = 4, P90 depicted) reporter mice as described in Fig. 1. Representative low-magnification overlay images of all channels show the PLVs with a highlighted region of interest (white boxes) (**A.a**,**B.a**). High-magnification images of the region of interest show Prox1 immunostain of LECs (**A.b**,**B.b**), αSMA immunostain of LMCs (**A.c**,**B.c**), Cre-driven tdT expression (**A.d,B.d**), overlay of all channels (**A.e**,**B.e**), and positive control BV VSMCs directly adjacent to PLVs (**A.f**,**B.f**). Note tdT expression (red) in PLVs (**A.d**,**B.d**) that colocalizes with αSMA^+^ LMCs (**A.e**,**B.e**), which is more profound (yellow) on the VSMC dense BVs, as expected (**A.f**,**B.f**).

To test whether PLV-LMCs emerge from VSMCs during neonatal development with constitutive Cre-drivers of VSMC progenitor markers, we utilized Prrx1^Cre^ and NG2^Cre^ drivers in our lineage tracing model at P21. For the NG2^Cre^ mice, we also assessed NG2-derived LMC contribution to PLVs after 6-weeks to confirm consistency of tdT expression compared to PLVs evaluated at P21. Our results demonstrate ubiquitous tdT expression in αSMA^+^ PLV-LMCs in Prrx1^Cre^ mice at P21 (Fig. 3A.a–e), and NG2^Cre^ animals at both P21 and after 6-weeks (Fig. 3B.a–e, P90 depicted). As expected, similar patterns were noted in the VSMCs of the adjacent blood vessel (BV) (Fig. 3A,B.f). This finding indicates that PLV-LMCs derive from a similar Prrx1^+^/NG2^+^ progenitor to VSMCs prior to P21.

### PLV-LMCs derive from a unique Prrx1^+^ and NG2^−^ progenitor cell incorporated onto PLVs between P10 and P21

**Figure 4:**
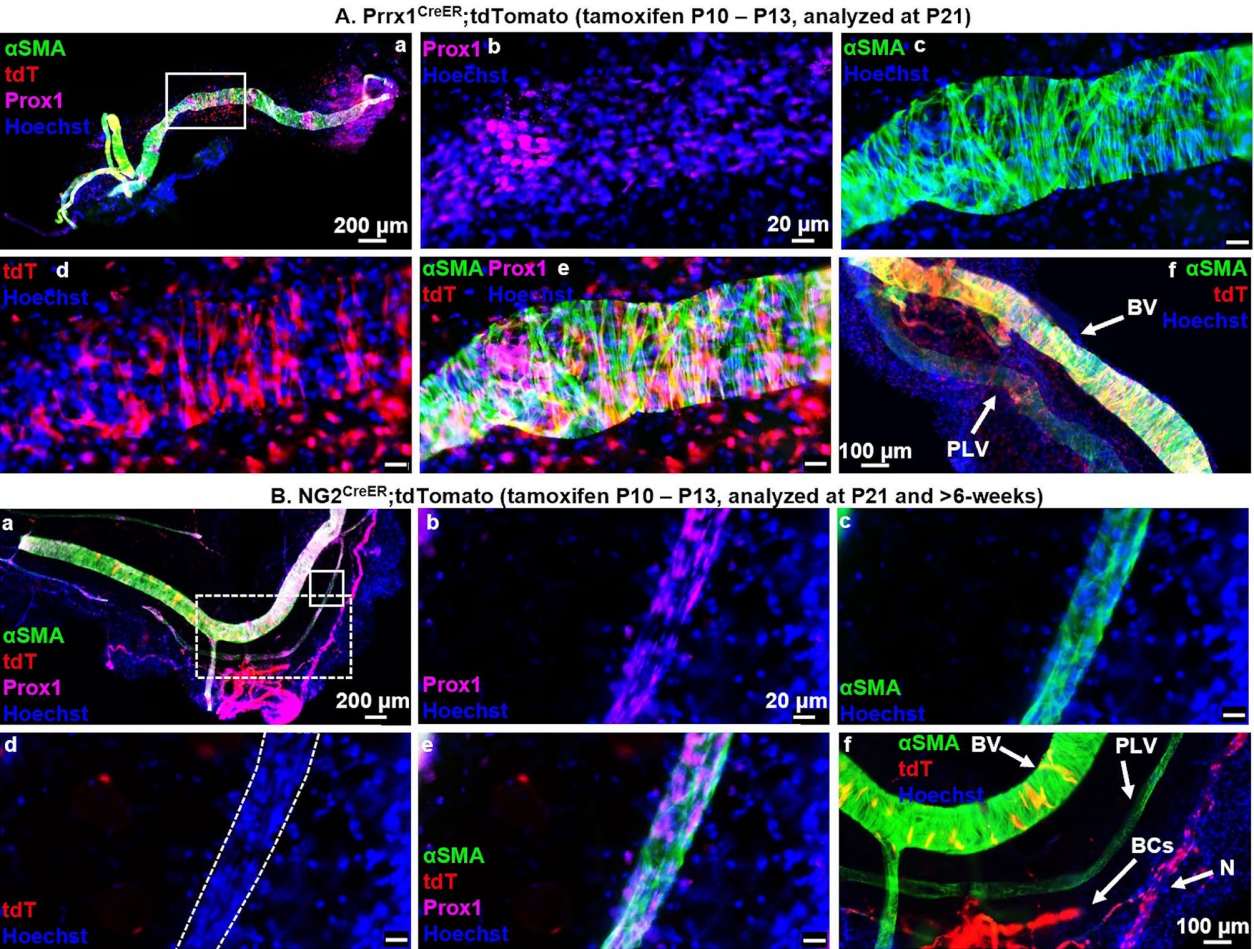
PLV-LMCs derive from a unique Prrx1^+^ and NG2^−^ progenitor cell incorporated onto PLVs between P10 and P21. Multicolor fluorescent microscopy was performed on whole mount immunostained PLVs and adjacent blood vessels (BVs) from tamoxifen-inducible Prrx1^CreER^ (n = 6, P21 depicted), and NG2^CreER^ (n = 5, P90 depicted) reporter mice as described in Fig. 1. Representative low-magnification overlay images of all channels show the PLVs with a highlighted region of interest (white boxes) (**A.a**,**B.a**). High-magnification images of the region of interest show Prox1 immunostain of LECs (**A.b**,**B.b**), αSMA immunostain of LMCs (**A.c**,**B.c**), Cre-driven tdT expression (**A.d,B.d**), overlay of all channels (**A.e**,**B.e**), and positive control BV VSMCs (**A.f**,**B.f**), blood capillary (BC) pericytes, and peripheral nerves (N) all directly adjacent to PLVs (**B.f**). Lineage tracing of Prrx1^CreER^ (**A.a**–**f**) mice demonstrates tdT expression in PLVs (**A.d**) that colocalizes with αSMA^+^ LMCs (**A.e**) along with successful Cre-recombination with tdT expression in positive control VSMCs, as expected (**A.f**). NG2^CreER^ (**B.a**–**f**) mice show absent tdT expression in PLVs (no red fluorescence in outlined PLV) (**B.d**) with successful tamoxifen induced NG2-driven Cre-recombination depicted by tdT expression in BV VSMCs, BC pericytes, and peripheral nerves (N), as expected (**B.f**). Note the few yellow VSMCs on the BV, which indicates that these cells were added to the fully formed BV after tamoxifen treatment.

After determining that PLV-LMCs and VSMCs may derive from a common source sometime prior to P21, we assessed whether this similarity was consistent throughout neonatal development by evaluating LMC recruitment to PLVs after P10. To study this specific timeframe during late neonatal growth and remodeling, we utilized tamoxifen-inducible CreER-driven lineage tracing models for Prrx1 and NG2. For both models, we induced with tamoxifen from P10–P13 with dissection of PLVs at P21. We also visualized the contribution of PLV-LMCs after 6-weeks in NG2^CreER^ animals to validate the results of the earlier time point (Fig. 1A.c). Our results show tdT expression colocalizing with αSMA^+^ LMCs in Prrx1^CreER^ PLVs (Fig. 4A.a–e), along with the VSMCs of the adjacent BVs (Fig. 4A.f). These findings promote the idea that these newly incorporating αSMA^+^/tdT^+^ PLV-LMCs in Prrx1^CreER^ animals also derive from a similar mesenchymal progenitor cell to VSMCs after P10. Similarly, the αSMA^+^/tdT^−^ PLV-LMCs represent LMCs that contributed to the PLV before P10, and also originated from a Prrx1-expressing progenitor cell based on our findings depicted in Fig. 3A. However, there was no tdT expression noted in αSMA^+^ LMCs of NG2^CreER^ PLVs at P21, or after 6-weeks, with standard tamoxifen induction from P10 to P13 (Fig. 4B.a–e, P90 depicted). Importantly, VSMCs, pericytes surrounding blood capillaries (BCs), and peripheral nerves (N) demonstrated successful NG2-driven Cre-recombination with clear tdT expression directly adjacent to the NG2^−^ LMCs of the PLV (Fig. 4B.f). As an additional indication of appropriate tamoxifen efficiency, glial cells in the brains of NG2^CreER^ mice at P21 demonstrated profound NG2-driven tdT expression, as expected (Supplementary Figure S1). Biostatistical analysis further validated the NG2-negativity by assessing relative Prrx1 contribution to PLV-LMCs, which determined a 9.78 × 10^−27^% chance of having missed a potential NG2^+^ LMC if these progenitors existed after P10 (Supplementary Figure S2, calculation outlined in Methods). Additionally, in vivo BrdU labeling confirmed consistent cellular contribution to PLVs with 26.15 ± 9.38 BrdU^+^ cells (LMCs and LECs)/mm of PLV/day during the postnatal period from P13 to P33 (assuming LMCs are terminally differentiated) (Supplementary Figure S2). Thus, neonatal PLV-LMCs derive from a unique Prrx1^+^/NG2^−^ muscle progenitor cell divergent from typical VSMC progenitors after P10.

## Discussion

To our knowledge, this is the first study focusing on the developmental lineage of LMC progenitors. Our results demonstrate that PLV-LMCs derive from two distinct origins during neonatal development. Prior to P10, PLV-LMCs originate from Prrx1^+^/NG2^+^ progenitors similar to VSMCs, while after P10 during later neonatal growth PLV-LMCs diverge from this common pathway and derive from unique Prrx1^+^/NG2^−^ progenitor cells. We also discovered that PLV-LMCs do not develop from typical skeletal muscle progenitors with Pax7- and MyoD-negativity, despite their striated muscle phenotype^6^. Thus, we conclude that PLV-LMCs derive from Pax7^−^/MyoD^−^/Prrx1^+^/NG2^+^ progenitors sometime during embryonic and/or early neonatal development before P10, but by P10 newly contributing PLV-LMCs originate from a transcriptionally distinct Pax7^−^/MyoD^−^/Prrx1^+^/NG2^−^ progenitor.

**Figure 5:**
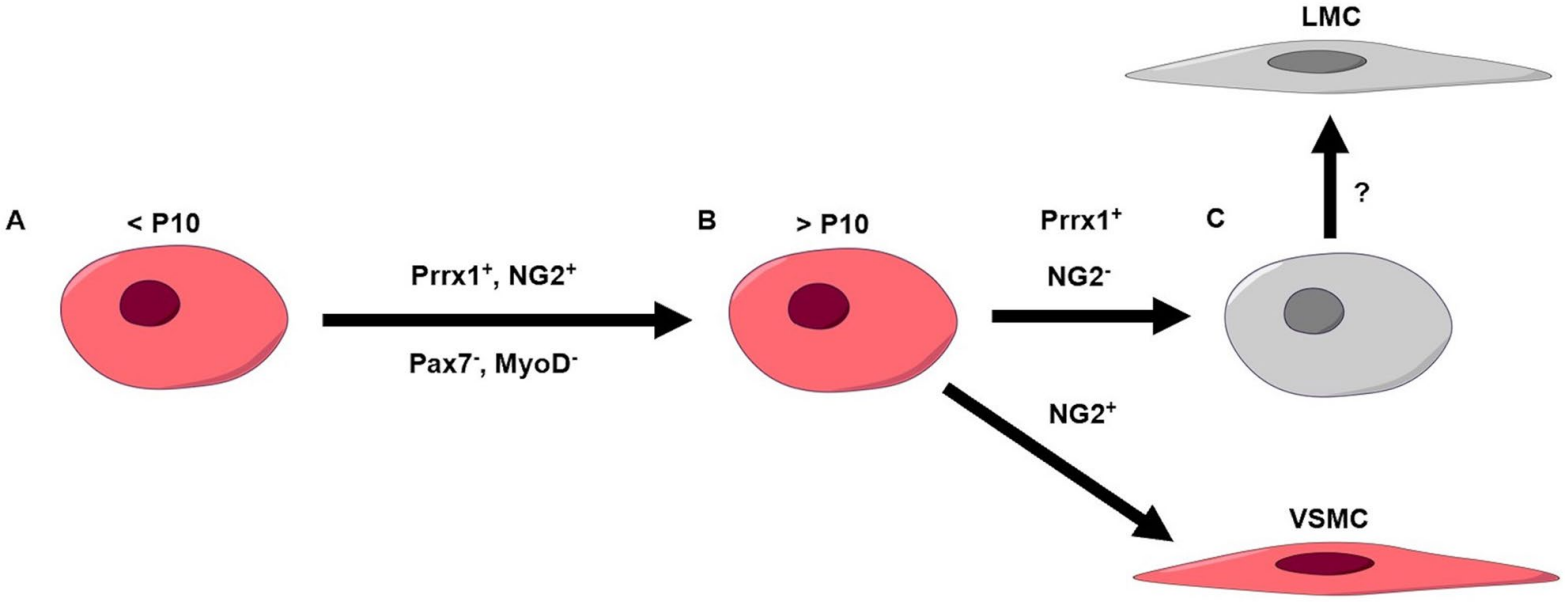
Neonatal PLV-LMC progenitor cell pathways. Prior to P10, LMCs derive from a Pax7^−^/MyoD^−^/Prrx1^+^/NG2^+^ progenitor similar to VSMCs (**A**). Between P10 and P21, newly incorporating PLV-LMCs diverge from the similar Prrx1^+^/NG2^+^ VSMC progenitor pathway and derive from distinct Prrx1^+^/NG2^−^ progenitors (**B**). These Prrx1^+^/NG2^−^ PLV-LMC progenitors likely utilize currently unknown transcriptional switches to specify their incorporation to the PLV (**C**).

While the differential expression of NG2 during PLV-LMC development indicates that PLV-LMC progenitors derive from two distinct sources, the ubiquitous expression of tdT in PLV-LMCs of NG2^Cre^ animals at both P21 and after 6-weeks makes this discovery difficult to understand. If PLV-LMCs are originating from a distinct NG2^−^ progenitor cell pool by P10, then we would expect tdT expression in NG2^Cre^ PLV-LMCs to decrease over time, which we did not observe in our model. However, this impression is only true if PLV-LMCs were continually being maintained by a progenitor cell that never expressed NG2. Alternatively, NG2^−^ PLV-LMC progenitors may originate directly from an NG2^+^ cell population, such as NG2^+^ pericytes. NG2^+^ pericytes are known to surround blood capillaries and provide structural integrity to larger blood vessels, while these tissue resident cells also serve as a progenitor cell population for VSMCs^22–29^. Our data suggests that NG2^+^ pericytes do not contribute to LMCs after P10, but LMCs may originally derive from a common NG2^+^ pericyte precursor to VSMCs. We therefore hypothesize that there is a late-stage NG2^−^ muscle progenitor cell specific for LMCs that originates from early NG2^+^ progenitors (Fig. 5A), with transcriptional silencing of NG2 sometime before P10 (Fig. 5B).

For future studies, the tissue localization of LMC progenitors remains an important question. Based on preliminary findings from these lineage tracing studies, we believe that quiescent PLV-LMC progenitor cells reside in the perivascular interstitium and adipose tissue adjacent to PLVs. Once these perivascular progenitors are activated, they require particular transcriptional switches that do not involve NG2 in order to add specifically to the lymphatic vasculature (Fig. 5C). In support of this hypothesis, we observed tdT^+^ perivascular cells with central Hoechst^+^ nuclei present in our Prrx1^Cre^ (Fig. 3A.d), NG2^Cre^ (Fig. 3B.d), and Prrx1^CreER^ (Fig. 4A.d) models, which were notably absent in the perivascular region surrounding NG2^CreER^ PLVs (Fig. 4B.d). Thus, the myogenic potential of these perivascular cells, and the signals that specify LMC progenitors to the lymphatic vasculature, warrant elucidation in future studies.

A limitation of our study is that we did not evaluate an exhaustive array of progenitor cell markers involved in VSMC and skeletal muscle cell development. Nevertheless, we are able to conclude that, by P10, PLV-LMC progenitors derive from a source that is transcriptionally distinct from the typical differentiation pathways of VSMCs and skeletal myocytes. However, the possibility that there may be subsets of either VSMCs or skeletal myocytes that also originate from Pax7^−^/MyoD^−^/Prrx1^+^/NG2^−^ progenitors similar to LMCs remains open. In addition, LMC progenitors may express other markers not assessed in this work that could more specifically define their relationship to the origin of these muscle cell types (i.e. for skeletal myocytes, Pax3 or Myf5; for VSMCs, APLNR or PDGFRα). Future studies building off this work will more specifically define the transcriptional niche of LMCs within the differentiation pathways of known muscle progenitors.

While we have focused specifically on PLVs in this study, the origin of LMCs in particular lymphatic beds (i.e. mesenteric, dermal, etc.) may represent entirely distinct progenitor cell populations as has been noted for VSMCs^23^. In fact, mesenteric LMCs demonstrate unique contractile physiology compared to LMCs in other tissues, which provides precedence for multiple LMC progenitor origins^4,30^. Previous studies also indicate that the CLVs in specific tissues may exhibit distinct LMCs based on protein expression. After P14, dermal LMCs are NG2^−^^24^, while NG2^+^ mural cells were apparent by embryonic day 17.5 (E17.5) surrounding mesenteric CLVs assessed by immunofluorescent staining^31^. Importantly, dermal LMCs do not contribute to the CLV until P14, and mesenteric LMCs begin developing during embryogenesis, similar to PLVs. Thus, these markers of LMCs noted previously in particular tissues may not be a product of the tissue specificity itself, but instead may indicate the progenitor cell population from which the LMCs originate based on the stage of development (i.e. before or after P10). However, the specificity of LMC progenitors in particular tissues remains an open question that warrants further investigation based on the distinct contractile physiology of LMCs throughout the body.

Despite our novel findings of PLV-LMC contribution during normal development, LMC progenitors that replenish functional LMCs and restore lymphatic contractions may derive from an entirely different source. For this reason, it is important that future studies determine the origin of LMC progenitors in the context of LMC injury, and the mechanisms that dictate their myogenic potential. In fact, the type of injury inflicted on LMCs may determine the regenerative capacity of LMC progenitors. While we have noted successful recovery of lymphatic contractions following anti-TNF therapy in mice with inflammatory arthritis^11^, other types of PLV-LMC injury have indicated relatively ineffective LMC regeneration. For instance, PLV-LMCs are extensively lysed by *Staphylococcus aureus* both in vivo and ex vivo as a mechanism of PLV dysfunction following skin and soft tissue infections^32^. In this model, however, the regeneration of PLV-LMCs is relatively slow and never fully reinstates PLV function. Thus, to fully understand LMC regeneration in particular disease states, consideration of different mechanisms that regulate the contribution of LMC progenitors to CLVs, such as cellular arrest or progenitor cell depletion, is essential.

In conclusion, we have taken an initial step towards defining the origins of PLV-LMC progenitors and determining their myogenic mechanisms. Importantly, we have established that, at some point during early neonatal development, PLV-LMCs begin deriving from a unique Pax7^−^/MyoD^−^/Prrx1^+^/NG2^−^ progenitor cell distinct from the differentiation pathways of known muscle progenitors. This discovery provides promise that specifically targeting LMCs and their progenitors will be possible in the near future. In addition, elucidating the comprehensive transcriptional profile of LMC progenitors provides a unique approach for identifying the elusive LMC-specific markers crucial for future studies of LMC function in vivo. The characterization of LMC progenitors will undoubtedly identify novel therapeutic potential for diseases with lymphatic dysfunction, such as inflammatory arthritis. By accomplishing these goals, we will catalyze the expansion of pivotal research in lymphatic biology.

## Methods

### Ethical approval

All murine studies were performed on protocols approved by The University of Rochester Committee for Animal Resources, within an AAALAC accredited vivarium. All experiments were performed in accordance with the associated guidelines and regulations for working with live vertebrate animals. Thus, our animal protocols comply with the animal ethical principles under which *Scientific Reports* operates.

### Mouse models

Forty male and female C57BL/6J mice (except MyoD^iCre^ on an FVB/NJ background, crossed into C57BL/6J for one generation) older than 3-weeks were used for this study. No animals died unexpectedly or were excluded. The following animals were used in the study: MyoD^iCre^ (a gift from Dr. David Goldhamer, University of Connecticut; Jackson Laboratory 014140, n = 3), Pax7^Cre^ (Jackson Laboratory #010530, n = 7), NG2^Cre^ (Jackson Laboratory #029926, n = 4), NG2^CreER^™ (NG2^CreER^, Jackson Laboratory #008538, n = 5), Prrx1^Cre^ (Jackson Laboratory #005584, n = 3), and Prrx1^CreERT2-GFP^ (Prrx1^CreER^, Jackson Laboratory #029211, n = 6) all crossed into Ai9^tdTomato^ reporter animals (Jackson Laboratory #007909). For sample sizes, “n” refers to number of animals throughout the manuscript unless otherwise stated (sample size specifics are provided in Supplementary Table S1). All of the images provided demonstrate PLVs from a single animal as representative from the group. Importantly, PLV-LMC contribution assessed at P21 or after 6-weeks was not notably different in any group and thus sample size for each strain was determined using both time points.

Generation of the reporter mice is summarized in Fig. 1A.a,A.b. Briefly, all double-transgenic experimental animals were heterozygous for the gene-specific Cre (+/−) and the Ai9^tdTomato^ reporter transgene (+/−), while the single-transgenic negative control animals were Cre-negative (−/−) and heterozygous for the Ai9^tdTomato^ transgene (+/−) (Supplementary Figure S3 depicts Cre-negative data). Of note, the fluorescence generated by the Ai9^tdTomato^ reporter is not specific to a cellular compartment and is expected to be visualized in both the cytoplasm and the nucleus. Importantly, Prrx1^CreERT2-GFP^ GFP fluorescence was assessed with and without addition of AF488-conjugated αSMA antibodies. The αSMA antibody was much brighter than the Prrx1-driven GFP fluorescence, and thus the αSMA antibody was applied and the GFP Prrx1 signal could not be detected at the optimum exposure for αSMA imaging.

Most of the Cre lines were retained as heterozygotes, and genotyping was performed using the following primer sequences: 5′-CCT GGA AAA TGC TTC TGT CCG TTT GCC-3′ (Cre Forward); 5′-GAG TTG ATA GCT GGC TGG TGG CAG ATG-3′ (Cre Reverse). Pax7^Cre^, Prrx1^CreER^, and Ai9^tdTomato^ lines were maintained as homozygotes as the recommended breeding system. For pairing of experimental animals, all Cre lines were males, while the Ai9^tdTomato^ animals were the females. Both male and female mice were used experimentally, and all groups were sacrificed at 3-weeks of age. Additional mice were sacrificed at or after 6-weeks of age for all gene markers with absent tdT expression on LMCs at any point during neonatal development to confirm that the negative results could not be explained by the timeframe analyzed. Tamoxifen induction was performed daily from P10 to P13 with 0.1 mg/g intraperitoneal tamoxifen (Millipore Sigma Cat# T5648, note chemotherapeutic hazard) (Fig. 1A.c). 5-Bromo-2′-deoxyuridine (BrdU) administration was performed daily from P13 to P33 with 0.1 mg/g intraperitoneal BrdU (Millipore Sigma Cat# B5002, note carcinogenic hazard) (Supplementary Figure S2). Injections were administered using BD Safetyglide Insulin Syringes: 0.3 mL, 31G × 6 mm (BD Cat# 328449).

### Gene markers

For skeletal myogenesis, Pax7 and MyoD are early transcription factors directly involved in the development of skeletal myocytes^33–37^. While Pax7 and MyoD are dependent on each other during skeletal myogenesis, we could not assume that this relationship would exist similarly in the differentiation of unknown LMC progenitors. In addition, during development of VSMCs, Prrx1 is a transcriptional regulator expressed in pluripotent mesenchymal progenitor cells^22,38,39^. NG2 is a robust nervous system and pericyte marker present at lower levels in VSMCs during development and regeneration. Pericytes demonstrate limited contractility on small blood capillaries, and also contribute to the structural integrity of larger blood vessels. Importantly, pericytes have been shown to serve as a progenitor cell population in the perivascular region surrounding blood vessels, identified by NG2-expression^22–29^.

### Tissue collection

The PLV dissection procedure is outlined in Fig. 1B and in the Supplementary Video. Two PLVs (blue vessels; blue arrows in Fig. 1B) are present on either side of the saphenous vein. These dual PLVs are considered replicates of each other for this study, thus in a single animal we are able to dissect and image up to 4 different PLV replicates (2 per hindlimb).

### Immunofluorescent staining and whole mount microscopy

Upon explant, the PLVs and diaphragms were placed in 1 × PBS in 0.65 mL Eppendorf tubes. The vessels were then fixed with 10% neutral buffered formalin (NBF) for 30 min at room temperature (RT). Following three 10-min washes with 0.1% Triton X-100 (Millipore Sigma Cat# X100) in 1 × TBS (Bio-Rad Cat# 1706435), the PLVs were permeabilized with 0.5% Triton X-100 in 1 × TBS for 1 h at RT or 0.3% Triton X-100 in 1 × TBS overnight at 4 °C. If performing BrdU labeling, the PLVs were treated with 2 M hydrochloric acid (Millipore Sigma Cat# 320331) for 1 h at RT, neutralized with 0.1 M Pierce 20X Borate Buffer (Thermo Fisher Scientific Cat# 28341) for 30 min at RT, and washed three times for 10 min with 0.1% Triton X-100 in 1 × TBS. The PLVs were then blocked with 5% Normal Goat Serum (NGS; Thermo Fisher Scientific Cat# 50062Z), 0.3% Triton X-100 in 1 × TBS for 1 h at RT. After blocking, primary antibodies were diluted in 5% NGS, 0.3% Triton X-100 in 1 × TBS and applied to the PLVs overnight at 4° C. Following three 10-min washes with 0.1% Triton X-100 in 1 × TBS, the secondary antibody was diluted in 5% NGS, 0.1% Triton X-100 in 1 × TBS and applied to the PLVs for 2 h at RT. The tissue was then washed three times for 10 min with 0.1% Triton X-100 in 1 × TBS and whole mounted using ProLong Diamond Antifade Mountant (Thermo Fisher Scientific Cat# P36970) and one drop of NucBlue Live ReadyProbes Reagent (Thermo Fisher Scientific Cat# R37605) as the nuclear Hoechst 33342 stain. During the staining process, all tissue was covered in tinfoil to block light that may interfere with the antibody or genetic fluorescence. The tissue was then imaged by VS120 Slide Scanner epifluorescent and/or Nikon A1R HD/Olympus FV1000 confocal microscopy (Fig. 1C). Cre-negative tissue was similarly processed and compared to the Cre-positive experimental tissue to ensure accurate identification of Cre-driven tdT reporter expression instead of autofluorescent tissue or aberrant reporter leak (Supplementary Figure S3).

Brains were drop-fixed in a 15 mL Falcon tube filled with 10% NBF for 48 h at 4 °C before being transferred to 1 × PBS. The brains were then sliced into 100 μm sections in the coronal plane and mounted with Hoechst as described above (Supplementary Figure S1).

### Antibodies

The following antibodies were used in this study: Rabbit anti-Prox1 (AngioBio Cat# 11-002P, 1:100 dilution), Mouse anti-αSMA Alexa Fluor 488 Conjugate (Abcam Cat# ab184675, 1:200 dilution; Thermo Fisher Scientific Cat# 53-9760-82, 1:100 dilution), Rat anti-BrdU Alexa Fluor 647 Conjugate (Abcam Cat# ab220075, 1:100 dilution), and Goat anti-Rabbit IgG DyLight 650 Conjugate (Thermo Fisher Scientific Cat# SA5-10034, 1:400 dilution).

### Image analysis

To establish BrdU^+^ cell contribution to PLVs, 200 × confocal stacks were generated along the entire length of a PLV. We assessed thresholded BrdU pixel area in Fiji^40^. By comparing the pixel area generated in Fiji and manual BrdU^+^/Hoechst^+^ cell counts in select images using QuPath^41^, we determined an estimated cell count. These cell counts were then compared to total length measures using QuPath to calculate cells/mm PLV/day, which includes both incorporating LECs and LMCs (Supplementary Figure S2).

### Statistics

For confirming NG2 negativity (Supplementary Figure S2), we utilized the following analysis. Using average estimated muscle cell counts in Prrx1^CreER^ BVs (n = 4 BVs) and PLVs (n = 10 PLVs), an expected ratio of VSMCs to LMCs was generated following tamoxifen induction from P10 to P13 and sacrifice at P21 (58.73 VSMCs/28.17 LMCs = 2.08 VSMCs/LMC in a 200 × HPF with Prrx1^+^ muscle cells). From this ratio, it was determined that, if LMCs from NG2^CreER^ animals were actually NG2^+^, then the average estimated muscle cell counts in NG2^CreER^ BVs (n = 5 BVs) leads to an expectation of ~ 3.55 NG2^+^ LMCs in a 200 × HPF of a PLV (7.40 NG2^+^ VSMCs/2.08 Prrx1^+^ VSMCs/LMC = 3.55 NG2^+^ LMCs). However, there are 0 NG2^+^ LMCs noted in all NG2^CreER^ PLVs (n = 17 PLVs). Since 87.4% of Prrx1^+^ LMCs are already expected to be NG2^−^ in these models ([1 − (3.55 expected NG2^+^ LMCs/28.17 Prrx1^+^ LMCs)] * 100 = 87.4%), there are 28.17 Prrx1^+^ LMCs per HPF, and 17 NG2^CreER^ PLVs were analyzed, the chance of having missed an NG2^+^ LMC is 9.78 × 10^−27^% ([0.874^28.17^)^17^ * 100 = 9.78 × 10^−27^%). Only 3-week-old Prrx1^CreER^ BVs and PLVs were used, while both 3-week- and 12-week-old NG2^CreER^ BVs and PLVs were used because no changes in muscle cell coverage were noted between the two time points (BVs: ~ 7–9 NG2^+^ VSMCs/HPF; PLVs: 0 NG2^+^ LMCs/HPF). One Prrx1^CreER^ BV was excluded due to an imaging artifact.

## Supplementary information

Supplementary Video.

Supplementary Information.

## Data Availability

The data generated and/or analyzed during the current study are available from the corresponding author on reasonable request.
