# Supplementary material for: Lineage tracing reveals evidence of a popliteal lymphatic muscle progenitor cell that is distinct from skeletal and vascular muscle progenitors
Source: Sci Rep. 2020 Oct 22;10:18088. doi: 10.1038/s41598-020-75190-7 (PMC7581810; doi:10.1038/s41598-020-75190-7)
Supplement: Supplementary file 2 — Supplementary Information. [file 41598_2020_75190_MOESM2_ESM.pdf]

Supplementary Information

Title

Lineage Tracing Reveals Evidence of a Popliteal Lymphatic Muscle Progenitor Cell that is Distinct from Skeletal and Vascular Muscle Progenitors

Authors

H. Mark Kenney[1,2], Richard D. Bell[1,2], Elysia A. Masters[1,3], Lianping Xing[1,2], Christopher T. Ritchlin[1,4], Edward M. Schwarz[1,2,3,4,5]

[1] Center for Musculoskeletal Research, [2] Department of Pathology, [3] Department of Biomedical Engineering, [4] Division of Allergy, Immunology, Rheumatology, Department of Medicine, [5] Department of Orthopaedics, University of Rochester, Rochester, NY, USA

Supplementary Table S1

Group	3w (N = # Animals)	>6w (N = # Animals)	Total (N = # Animals
**Pax7^{Cre}**	N = 4	N = 3	N = 7
**MyoD^{iCre}**	N = 2	N = 1	N = 3
**Prrx1^{Cre}**	N = 3	N = 0	N = 3
**NG2^{Cre}**	N = 1	N = 3	N = 4
**Prrx1^{CreER}**	N = 6	N = 0	N = 6
**NG2^{CreER}**	N = 2	N = 3	N = 5
**Cre/ER-Negative**	N = 7	N = 5	N = 12
**Total**	N = 25	N = 15	N = 40

Supplementary Table S1. Number of animals with PLVs analyzed for each strain and time point.

Supplementary Figure S1

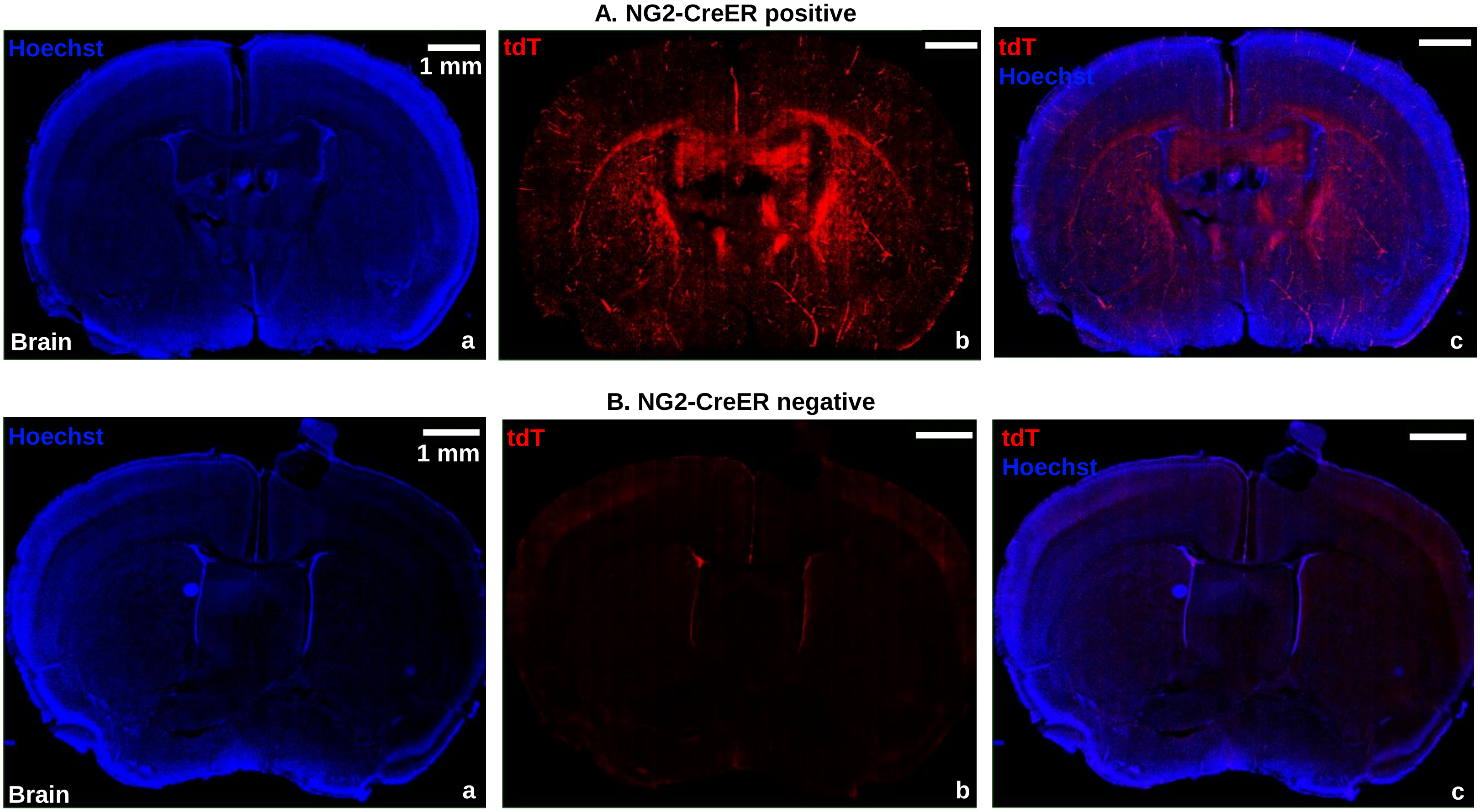

Supplementary Figure S1. tdT+ glial cells in the brains of NG2^{CreER} animals demonstrates efficient tamoxifen induction. Fluorescent microscopy of coronally sliced brains from P21 NG2^{CreER} animals induced with tamoxifen from P10 – P13 demonstrates the nuclear architecture of the brain **(A.a)** with expected NG2-driven tdT expression in glial cells **(A.b)** further demonstrated in the Hoechst/tdT overlay **(A.c)** as NG2 positive control tissue. To ensure true NG2-driven tdT signal, a Cre-negative control was analyzed showing the similar nuclear architecture of the brain **(B.a)** without notable tdT expression **(B.b)** further demonstrated with the Hoechst/tdT overlay **(B.c)** indicating reporter integrity. These control tissues highlight the successful and efficient NG2-driven tdT expression using the tamoxifen induction outlined in Fig. 1A.c in the NG2^{CreER} animal model.

Supplementary Figure S2

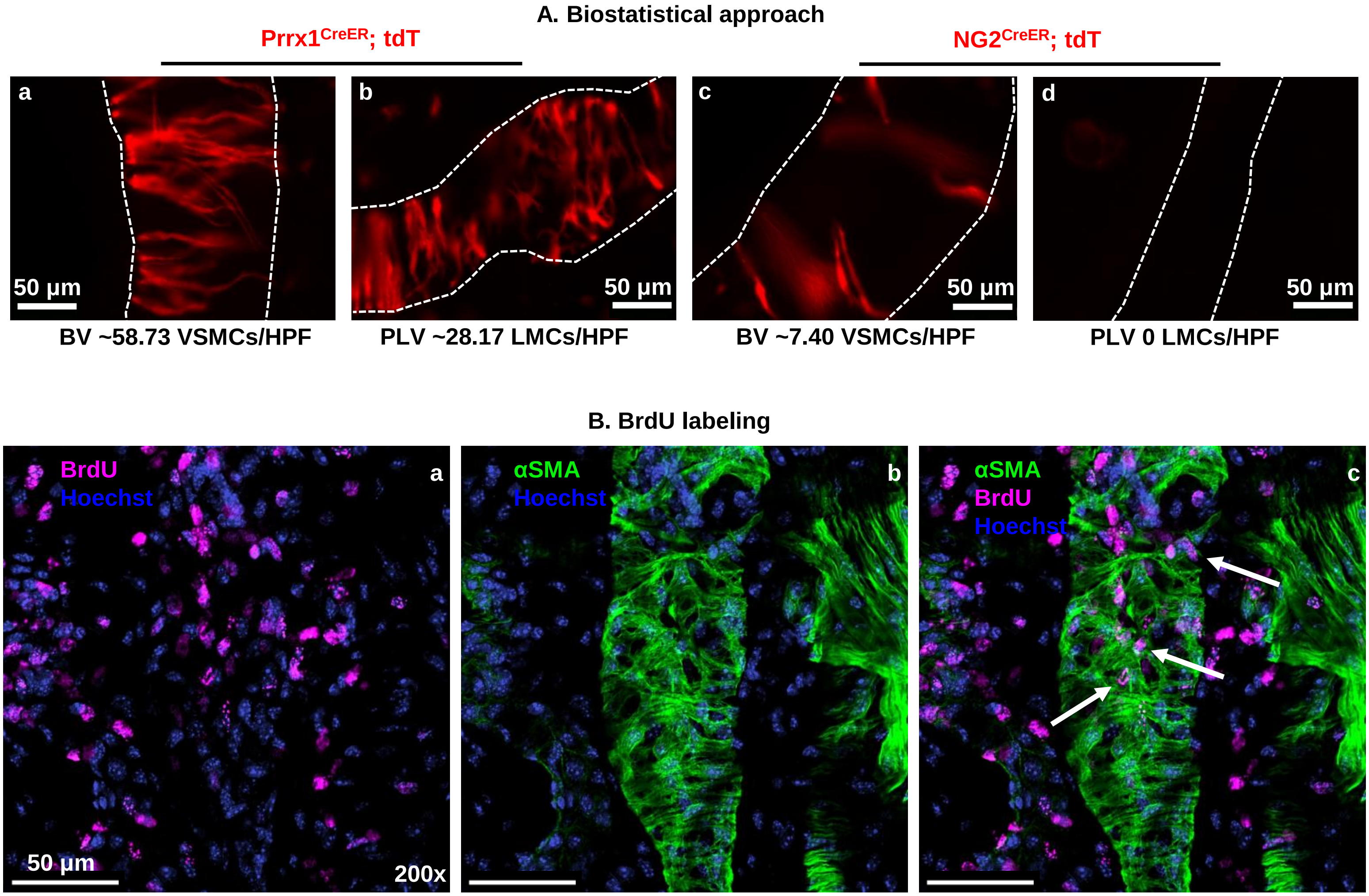

Supplementary Figure S2. Dynamic labeling confirms *de novo* LMCs in PLVs of mice are not derived from NG2$^+$ progenitors after P10. Prrx1^{CreER} x tdT (n = 6 PLVs, n = 4 BVs at P21), and NG2^{CreER} x tdT (n = 17 PLVs, n = 5 BVs total) reporter mice were treated with tamoxifen on P10 – P13, and PLVs and adjacent BVs were harvested on P21 (Prrx1 and NG2) or P90 (NG2) for direct fluorescent microscopy as described in Fig. 1. There was no change in tdT$^+$ cells in NG2^{CreER} mice between P10 – P90 validating both time points for analysis (~7 – 9 tdT$^+$ VSMCs / HPF, 0 tdT$^+$ LMCs / HPF). Representative fluorescent images of lineage traced cells (red) in vessels (highlighted by white dashed lines) of a Prrx1$^+$ BV **(A.a),** Prrx1$^+$ PLV **(A.b),** NG2$^+$ BV **(A.c**, P90 depicted**),** NG2$^+$ PLV **(A.d**, P90 depicted**)** are shown. Based on the relative contribution of Prrx1$^+$ VSMCs **(A.a)** to Prrx1$^+$ LMCs **(A.b)** compared to NG2$^+$ VSMCs **(A.c)**, we determined that the statistical probability of missing an NG2$^+$ LMC **(A.d)** was 9.78 x 10^{-27}%, thus confirming that LMCs after P10 are derived from NG2$^-$ progenitors. To confirm *de novo* LMC incorporation into PLVs during the lineage tracing study period, mice received intraperitoneal BrdU daily from P13 until euthanasia on P33, and their PLVs were harvested for Hoechst-staining (blue) and multicolor whole mount immunofluorescent microscopy using AF647-conjugated antibodies against BrdU (purple) and AF488-conjugated antibodies against αSMA to mark LMCs (green). Representative images of the BrdU$^+$ cells **(B.a)**, αSMA$^+$ LMCs **(B.b),** and merged image **(B.c)** are shown. Note the BrdU$^+$/αSMA$^+$ cells (white arrows), indicating that *de novo* incorporation of LMCs into the growing/remodeling PLV occurred during the study period. Quantification of these labeled PLVs (n = 4 PLVs, n = 2 animals) demonstrated a *de novo* incorporation rate of 26.15 ± 9.38 BrdU$^+$ cells / mm PLV / day.

Supplementary Figure S3

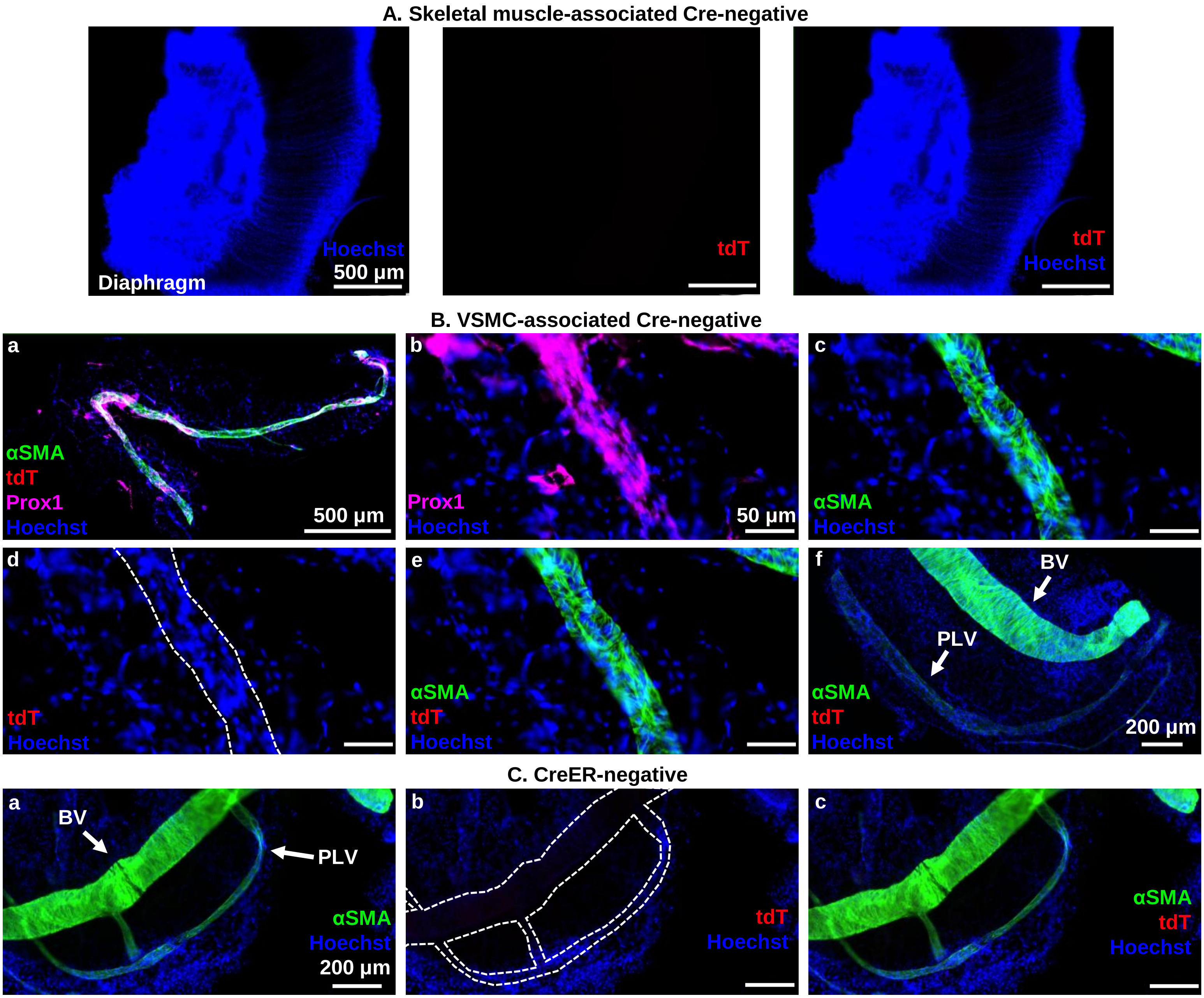

Supplementary Figure S3. Absent tdT fluorescence in Cre- and CreER-negative controls demonstrates Ai9 reporter integrity. Multicolor fluorescent microscopy was performed on whole mount immunostained PLVs from single transgenic Cre/CreER$^{-/-}$, Ai9$^{+/-}$ animals generated from crosses between Cre/CreER$^{+/-}$ and Ai9$^{+/+}$ mice as described in Fig. 1. Cre/CreER$^{-/-}$, Ai9$^{+/-}$ mice were analyzed at the same time as the experimental double transgenic Cre/CreER$^{+/-}$, Ai9$^{+/-}$ animals as negative controls (n = 12). CreER$^{-/-}$, Ai9$^{+/-}$ mice were similarly administered tamoxifen from P10 – P13. For Cre$^{-/-}$, Ai9$^{+/-}$ models of skeletal muscle progenitors, diaphragm skeletal myocytes outlined with nuclear Hoechst stain **(A.a)** demonstrate absent tdT expression **(A.b)**, which is further demonstrated by the Hoechst/tdT overlay **(A.c)**. Representative low-magnification images of a Cre$^{-/-}$, Ai9$^{+/-}$ PLV with a highlighted region of interest (white boxes) **(B.a)** is shown with corresponding high-magnification images of Prox1 immunostain of LECs **(B.b)**, αSMA immunostain of LMCs **(B.c)**, the Cre-driven tdT expression (note lack of red fluorescence within the white dotted lines that outline the PLV) **(B.d)**, and αSMA/tdT overlay **(B.e)**. Similarly, VSMCs lack red fluorescence in the Cre-negative control, as expected **(B.f)** (NG2^{Cre}-negative control depicted at P90 in contrast to the αSMA^{+}/tdT^{+} LMCs and VSMCs noted in the double transgenic Cre$^{+/-}$, Ai9$^{+/-}$ animals in Fig. 3). For the CreER$^{-/-}$, Ai9$^{+/-}$ negative control, a PLV and adjacent BV are shown immunostained for αSMA (**C.a)** with lack of tdT fluorescence following tamoxifen induction (note absent red signal within the white dotted lines that outline both the PLV and BV) **(C.b)**, which is further demonstrated in the αSMA/tdT overlay **(C.c)** (NG2^{CreER}-negative control depicted at P90 in contrast to the αSMA^{+}/tdT^{+} VSMCs noted in the double transgenic CreER$^{+/-}$, Ai9$^{+/-}$ animals in Fig. 4).
